# Current channeling along extended defects during electroreduction of SrTiO_3_

**DOI:** 10.1038/s41598-019-39372-2

**Published:** 2019-02-21

**Authors:** Christian Rodenbücher, Stephan Menzel, Dominik Wrana, Thomas Gensch, Carsten Korte, Franciszek Krok, Krzysztof Szot

**Affiliations:** 1 Forschungszentrum Jülich GmbH, Institute of Energy and Climate Research (IEK-3), 52425 Jülich, Germany; 2 Forschungszentrum Jülich GmbH, Peter Grünberg Institute (PGI-7), 52425 Jülich, Germany; 3 Forschungszentrum Jülich GmbH, JARA-FIT, 52425 Jülich, Germany; 4 Jagiellonian University, Marian Smoluchowski Institute of Physics, 30-348 Krakow, Poland; 5 Forschungszentrum Jülich GmbH, Institute of Complex Systems (ICS-4), 52425 Jülich, Germany; 6 University of Silesia, A. Chełkowski Institute of Physics, 40-007 Katowice, Poland

## Abstract

Electroreduction experiments on metal oxides are well established for investigating the nature of the material change in memresistive devices, whose basic working principle is an electrically-induced reduction. While numerous research studies on this topic have been conducted, the influence of extended defects such as dislocations has not been addressed in detail hitherto. Here, we show by employing thermal microscopy to detect local Joule heating effects in the first stage of electroreduction of SrTiO_3_ that the current is channelled along extended defects such as dislocations which were introduced mechanically by scratching or sawing. After prolonged degradation, the matrix of the crystal is also electroreduced and the influence of the initially present dislocations diminished. At this stage, a hotspot at the anode develops due to stoichiometry polarisation leading not only to the gliding of existing dislocations, but also to the evolution of new dislocations. Such a formation is caused by electrical and thermal stress showing dislocations may play a significant role in resistive switching effects.

## Introduction

Strontium titanate (SrTiO_3_) has become an intensively studied material. It is regarded as a model compound of dielectric oxides, offering a variety of interesting properties that can be exploited for energy-efficient applications. It can be used as a catalyst^1^, as an anode in solid oxide fuel cells (SOFC)^2^, as an oxygen sensor^3^ and has also become a promising material for novel electronic devices^4,5^. In particular, the resistive switching effect has attracted considerable attention, as it allows for the controlled manipulation of the oxide resistance by applying an electrical field. Such a phenomenon holds potential for the development of non-volatile memory or neuromorphic logic architectures^6–9^. In order to gain a fundamental understanding of the nature of resistive switching phenomena in metal oxides, electroreduction or electrodegradation experiments have become the method of choice in numerous research studies conducted in this field^10–14^. It was found that the underlying mechanism of the resistive switching effect relates to a local reduction process via the generation and movement of oxygen vacancies under an electric field, causing a valence change of the transition metal ion due to the electronic charge compensation^15–17^. In this way, the resistance of the oxide can be efficiently switched from insulating to semi-conducting, and even to metallic behaviour. Macroscopic electroreduction experiments have been reported, in which a single crystal or ceramic has been polarized by DC voltage, while the temporal evolution of the transformation has been monitored^10^. In particular, research performed with Fe-doped SrTiO_3_ has been found to show that the valence change leads to a significant change in the optical absorption from transparent, in the insulating state, to brown coloration in the reduced state^13,18^. It has therefore been concluded that electroreduction starts at the cathode and proceeds towards the anode, which can be regarded as movement of the virtual cathode^19,20^. On the basis of these investigations, models for resistive switching in nanoscale thin film devices have been developed and a reasonable agreement between experiment and modelling has been outlined^21–24^. However, before the widespread use of SrTiO_3_-based devices becomes viable, a variety of issues must be solved that demand a closer look at electroreduction phenomena on the nanoscale. It has already been demonstrated that by employing local-conductivity atomic force microscopy (LC-AFM), single dislocations can be resistively switched when addressing via the conducting AFM tip and sweeping the voltage, promising that memristive devices can, in principle, be scaled down to the ultimate limit, i.e., the size of a dislocation^25,26^. On the other hand, various experimental findings indicate that dislocations may also play a significant role for macroscopic electroreduction^25,27^. This is because during electroreduction, Joule heating is generated by the current flow through the oxide and temperature distribution can be used for the detection of conducting channels^28–30^. Thanks to the development of highly-sensitive thermal cameras, we are nowadays able to monitor the different stages of electroreduction with utmost precision. In this study, we perform experiments on a sample where locally the dislocation density has been increased by mechanical scratching in order to investigate the influence of dislocations. In comparison to reference experiments, we demonstrate that in the first stage of electroreduction the current is channelled along of areas with high dislocation density, while after prolonged electroreduction new dislocations are induced due to the electrical and thermal stress.

## Results and Discussion

**Figure 1:**
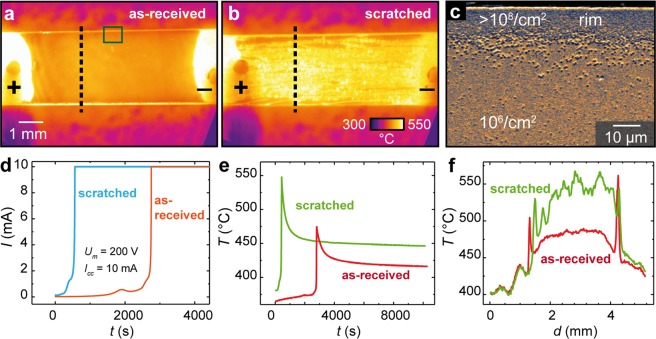
Electroreduction of SrTiO_3_ single crystals at *U*_*max*_ = 200 V. Thermal image of the: (**a**) *as-received*; and (**b**) scratched sample at the maximum temperature (at 450 s for the scratched and 2810 s for the polished sample); (**c**) electron micrograph (SEM) of the distribution of dislocations at the rim of a polished SrTiO_3_ crystal, marked by chemical etching in HF; (**d**) temporal evolution of the current; (**e**) temporal evolution of the mean temperature of the samples; (**f**) line profiles of (**a**) and (**b**) along the dashed line.

As is illustrated in Fig. 1, initially we compared the electroreduction of an epi-polished *as-received* SrTiO_3_ single crystal with a scratched crystal, namely a crystal where extended defects were introduced by roughening the surface using a diamond tip. Those extended defects can be assumed to consist mainly of dislocations or bundles of dislocations that are known to evolve upon the application of mechanical stress to the crystal^31^. As SrTiO_3_ does not possess a natural cleavage plane, the evolution of more ordered extended defects such as grain boundaries is not expected. This assumption is supported by TEM investigations showing the evolution of a hierarchical network of dislocations on the SrTiO_3_ surface after mechanical polishing^32^. Hence, a relatively broad dislocation-rich path is established via scratching intentionally making it possible to detect the influence of dislocations on the Joule heating effect optically. During the electroreduction experiment of the two crystals, the other parameters such as sample geometry, vacuum conditions, or applied voltage were kept constant. At the beginning of the experiment, a voltage of 200 V was applied via the Pt electrodes and was then reduced automatically when the current compliance of 10 mA was reached. The temporal plot of this macroscopic current shown in Fig. 1d illustrates this behaviour. Already here, the difference between the scratched and the polished surface gets striking. While the polished sample needed more than 40 minutes until breakdown occurred, the scratched sample gained high conductivity within a couple of minutes only. The thermal images (Fig. 1a,b) taken at the respective maximum temperatures following breakdown reveal that, in general, the temperature of the scratched sample was higher, which is additionally illustrated by the temporal plot displaying the average surface temperature calculated from the thermal images recorded during the electrodegradation process (Fig. 1e). This already gives a strong indication that dislocations have a significant impact on the electrical properties during electroreduction. Regarding the lateral distribution of the temperature, it can be seen that the maximum temperature of the *as-received* sample was reached at the rim of the sample, while at the scratched sample the temperature at the surface is significantly increased. Such observations become apparent in the temperature profiles shown in Fig. 1f, which are extracted from the thermal images as marked by the dashed lines in Fig. 1a,b. It can be seen that the temperature at the rim of the sample is up to 40 °C higher than in the centre of the polished region. This can also be explained by the influence of dislocations. During initial sample preparation, the *as-received* crystal was cut into smaller pieces (by diamond wire saw), introducing a high density of dislocations at the rims. This is confirmed by marking the dislocations by chemical etching using hydrofluoric acid, as can be seen in the electron microscope image of the etched surface in Fig. 1c. The density of etch pits is more than two orders of magnitude higher at the crystal rim (>10^8^/cm^2^) than on the epi-polished surface (10^6^/cm^2^). Hence, it can be assumed that the current in the first stage of electroreduction mainly flows along the rims due to the presence of dislocations.

**Figure 2:**
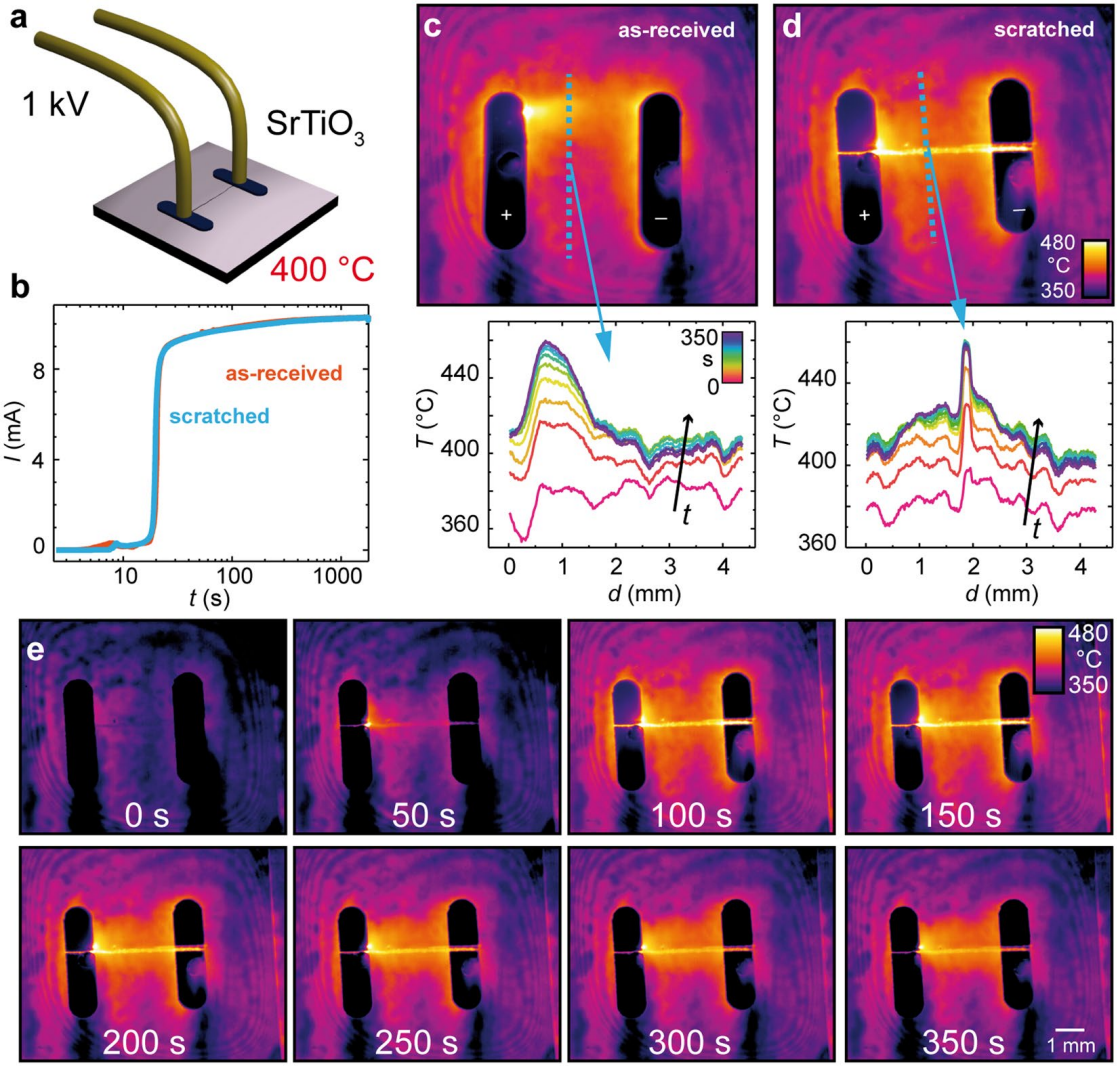
Geometrically-optimized SrTiO_3_ electroreduction experiment: (**a**) schematic illustration of the setup employing sputtered Pt electrodes; (**b**) macroscopically measured current through the oxide as a function of the degradation time at *U*_*max*_ = 1000 V. Thermal images and line profiles of the (**c**) polished; and (**d**) scratched samples at the maximum temperature (after approximately 100 s of electroreduction); (**e**) temperature evolution of the scratched sample; (**d**) showing the shift of maximum temperature from the scratch towards the hotspot region.

In order to have a closer look at the influence of artificially introduced dislocations, a more defined experiment was designed. Here, only one single scratch with a length of approximately 4 mm was generated in the centre of a 10 × 10 × 0.5 mm^3^ crystal. Subsequently, electrodes with a distance of 3 mm were deposited on top of the scratch by sputtering via a shadow mask. In order to avoid the current flows preferentially along the rims of the crystals, as in Fig. 1a, the electrodes were deposited with a distance of at least 2.5 mm to the rims, such that they do not touch them. Electric contacting within the vacuum chamber was realized by two metallic wires, as illustrated schematically in Fig. 2a. Likewise, in the previous experiments shown in Fig. 1, we compared the electroreduction of a scratched sample with an *as-received* epi-polished sample in the same geometry, using the same voltage of 1 kV. The current evolution as a function of time is presented in Fig. 2b, illustrating that the current increased exponentially within the first minutes of the experiment and saturated around 10 mA (the current compliance provided by the series resistor). Hence, the voltage drop over the oxide decreased from 1 kV up to a few volts during the course of electroreduction. In comparison to Fig. 1, the global difference between the scratched and polished sample is not as large, since only one single scratch was introduced, and also a higher voltage to accelerate the process was used. For the infrared images presented in a false colour plot in Fig. 2c,d, it can be clearly seen that the temperature along the scratch is significantly higher than that of its surrounding. This illustratively reveals, in line with the previous experiment in Fig. 1, that the current is channelled to regions with increased density of dislocations, causing local Joule heating. The time evolution of the temperature line profiles extracted from the temperature maps shows that due to Joule heating the base temperature increases by approximately 40 °C, but along the scratch an increase of more than 80 °C is observed. Laterally, the zone of increased temperature is confined to the scratch, indicating that the current density through the dislocation network induced by mechanical scratching is significantly higher than in the surrounding components.

To be sure that the observed temperature increase along the scratch is a real effect and not due to the geometrical effects of the scratched surface corresponding to a change in emissivity, for example, we conducted a reference experiment in which the scratched sample was heated externally by a ceramic heater. Here, only the average temperature increased but no significant local temperature increase at the scratch was observed, indicating that we indeed observed the current channelling along the scratching-induced dislocations. Regarding the polished sample (Fig. 2c), it can be seen that the temperature distribution was also not homogeneously distributed between anode and cathode. In fact, a hotspot at the anode evolved, having a temperature of around 500 °C and surrounded by a diffuse region of increased temperature. In general, a higher temperature at the anode region must be expected, since the ionic movement of negatively charged oxygen ions towards the anode would result in a stoichiometry polarization, whereupon the cathode becomes more reduced than the anode. Hence, the potential drop would be concentrated at the anode, resulting in a local increase in the Joule heating.

With respect to the time evolution of the temperature distribution of the scratched sample, as is shown in the thermal image sequence obtained at different times during electroreduction in Fig. 2e, the region with the highest temperature was shifted away from the scratch and the hotspot evolved a few hundred micrometres away at the rim of the anode when the electroreduction of the scratched sample proceeded (see the thermal image obtained at 150 s). At this later state of electroreduction, the scratch cooled down and the temperature difference between the scratch and surrounding components became less significant. Nevertheless, the temperature’s maximum was still close to the scratch region, indicating that after prolonged reduction, surrounding areas of the crystal are also reduced and the difference between scratched and unscratched crystal diminishes.

**Figure 3:**
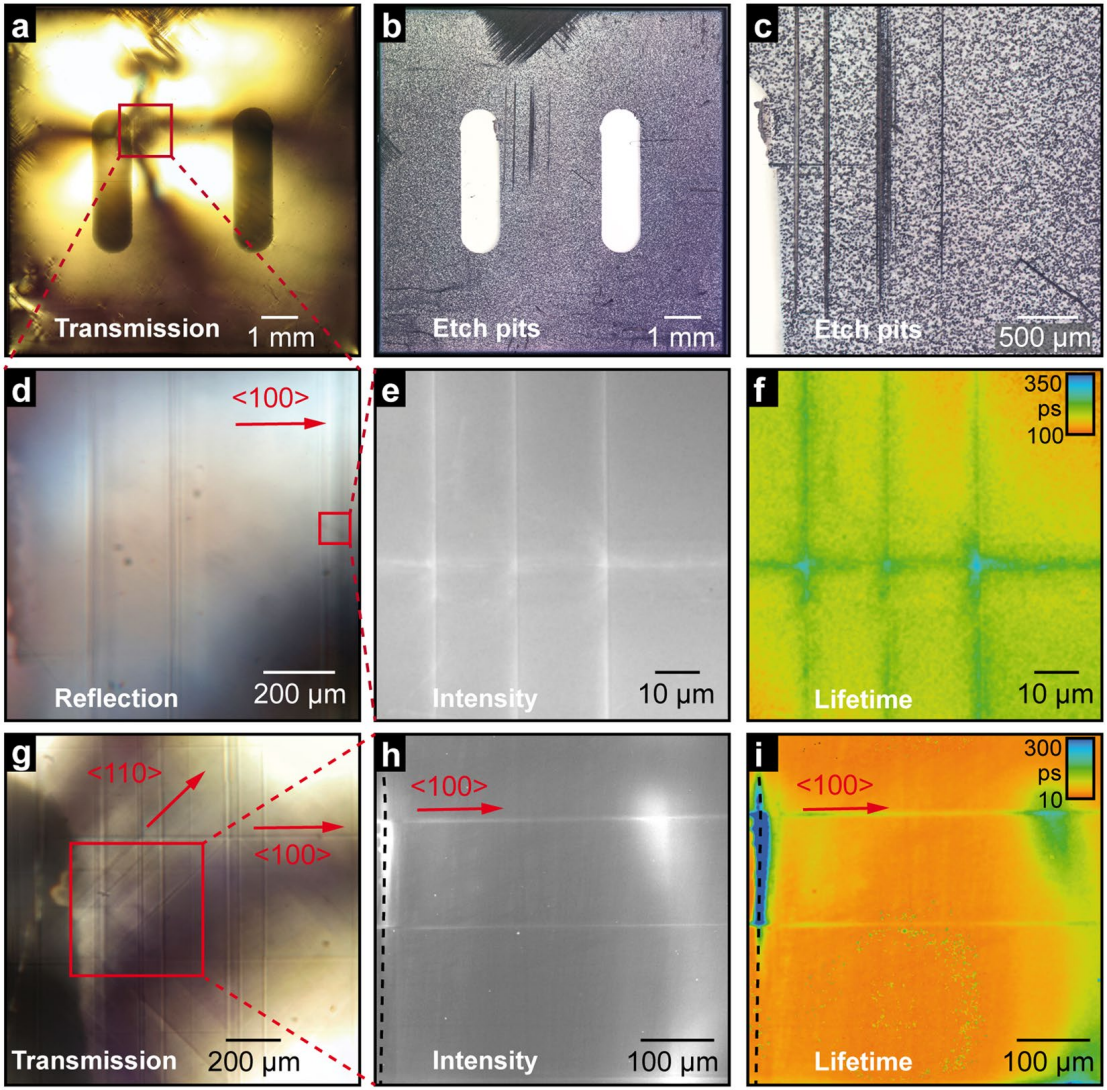
Investigation of the electroreduced polished sample from Fig. 2c: (**a**) optical phase contrast microscopy in the transmission light mode; (**b**) optical microscopy of the sample after etching in HF, displaying the exits of dislocations; (**c**) etch pits analysis; (**d**) phase contrast reflection-light microscopy of the hotspot region marked by the red square in (**a**); (**e**) fluorescence intensity; and (**i**) lifetime maps of the same region obtained by FLIM; (**g**) phase contrast reflection-light microscopy and fluorescence intensity; and (**i**) lifetime maps obtained in the region marked by the red box in (**g**) from deeper parts of the sample. The dashed lines in (**h**,**i**) mark the position of the anode.

Next, we analysed the impact of the prolonged electroreduction on various optical properties. Here, we focus on the characterization of the polished sample. Figure 3a shows optical microscopy images in transmission light mode and with phase contrast, illustrating the internal stress of the sample. Apparently, starting from the hotspot region, a significant distortion has spread through the crystal, which led to the generation of dislocations and movement via gliding, as confirmed by etch pit investigations (Fig. 3b,c). For this analysis, the electroreduced crystal had been etched in buffered hydrofluoric acid for 2 min at 90 °C. All etching investigations shown in this paper were performed after the electrical experiments. During the treatment by hydrofluoric acid, the exits of the dislocations are preferentially etched, allowing for the easy detection of dislocations. While in the major part of the crystal the dislocation density is typical for epi-polished Verneuil-grown crystals^33^, distinct bundles of dislocations aligned along the <100> crystal directions are present around the hotspot region. Also in the region, close to the edge of the crystal, where a high distortion was identified by phase contrast microscopy (Fig. 3a), a bundling of dislocations occurred, mainly along the <110> crystal axes (Fig. 3c). As we did not observe such large agglomerations of dislocations on comparable *as-received* samples and it has been shown that mechanically induced dislocations are preferentially aligned in <100> direction^34^, we suppose that the newly observed agglomeration in <110> direction evolved during electroreduction due to the high electrically induced stress (see details in supplement). According to Javaid *et al*., dislocation pile ups in <100> direction are related to dislocations lying in {110}_45°_ planes, while those in <110> direction correspond to dislocations in {110}_90°_ planes^31^. We have shown by investigating the bubble formation at the Pt/SrTiO_3_ boundary that locally a high oxygen pressure of more than 60 MPa can be induced electrically^13^. Hence it can be assumed that where regions with different ionic conductivities meet such as the already reduced hotspot and the pristine matrix, large mechanical stress can be present leading to the formation of additional dislocations even at temperatures as low as 500 °C. The dislocation-rich lines running in y-direction (perpendicular to the current flow) can also be identified using phase contrast microscopy in reflection (Fig. 3d, obtained before etching). Additionally, when measuring the same region in transmission light mode, pronounced lines in <100> direction, as well as in <110> can be seen close to the hotspot (Fig. 3g), indicating that a complex 3D network of dislocations had evolved during electroreduction. In those lines of high dislocation density, the electronic properties had also been significantly modified, as revealed by fluorescence-lifetime imaging microscopy (FLIM) (Fig. 3e,f,h,i), which is a proven method for the detection of local changes in electronic structure across the fluorescence lifetime^35^. In both, the intensity and fluorescence lifetime maps, it can be seen that two dislocation-rich lines along the <100> direction are distinctive. In Fig. 3e,f, perpendicular lines of the current flow are mainly mapped, which can be assumed to be closer to the surface due to their visibility in reflection-light mode (Fig. 3d). Respectively, in Fig. 3h,i, lines in deeper parts of the crystals were investigated by adjusting the focal plane of the FLIM setup. In these lines, the intensity of the fluorescence is significantly higher and the lifetime is longer than in the surrounding components. This indicates a higher amount of self-doping with oxygen vacancies along the lines, revealing that the electronic structure along the evolved conducting paths has been modified. Hence, in the state of prolonged reduction, the arrangement of dislocations may also influence the local transport properties significantly.

The role of dislocations as conducting channels can be understood by analysing the thermal reduction of SrTiO_3_ crystals. It has been calculated that the formation enthalpy of an oxygen vacancy at a dislocation is up to 2 eV lower than in the matrix^36^. Hence, in the first stage of reduction, dislocations are reduced preferentially, leading to the evolution of a conducting network. This model has also been supported experimentally by LC-AFM investigations showing that the first stage of reduction is related to a highly inhomogeneous surface conductivity with the exits of dislocations appearing as conducting spots within an insulating matrix^27^. Hence, we can assume that comparable effects are present during electroreduction, which is another means of reduction driven by electric field gradients. Therefore, when applying a high voltage to the insulating crystal, at first the dislocations will gain conductivity before the matrix will be reduced globally. The influence of dislocations on electroreduction and resistive switching is a highly complex phenomenon and other researchers, e.g. Kamaladasa *et al*.^34^, came to the conclusion that dislocations only have negligible impact on the electroforming behaviour related to resistive switching of sub-micron scale devices. However, the situation in our macroscopic experiment where we intentionally introduced a confined network of dislocations with high density and connectivity is different. As we have shown previously, oxygen is preferentially released from the dislocation-rich boundary of artificial SrTiO_3_ bi-crystals during electroreduction resulting in the localized bubble formation under the Pt electrode^32^. These experimental findings together with the theoretically predicted high reducibility^36^ of dislocation cores shows that the question of the role of dislocations during electroreduction is not as simple as previously thought and that artificially created dislocation structures can be used to define preferential conducting paths.

Now we want to illustrate the correlation between confined conducting paths and the surface temperature by finite element method simulations under the assumption that dislocations indeed act as current channels during electroreduction. As the original system has a quite large asymmetry in terms of length scale, which leads to too high computational cost, a model system with reduced dimensions is simulated. A slab of 10 µm length, 10 µm height and variable width *w* is considered. The slab contains exactly one conducting cylindrical filamentary region with a diameter of 20 nm, connecting the two electrodes. Such size was chosen based on the dimensions of the conductive spots seen in the conductivity and work function maps of the reduced SrTiO_3_ surfaces^27^. In the simulation model, the filament is located 50 nm below the top surface of the sample. The static heat equation is as follows^37^:

1 $$\,{\rm{\nabla }}{k}_{th}{\rm{\nabla }}T=-\,JE,$$

while the current continuity equation is^38^:

2 $$\nabla J=\nabla \sigma \nabla \phi =0$$

and these are self-consistently solved for the temperature *T* and potential *φ*. In Eq. (1), *J* is the current density and *E* is the electric field, which can be calculated from the potential *φ*. The thermal conductivity is assumed to be constant within the entire geometry, i.e., *k*_th_ = 8 W/mK^39^. The electric conductivity of the filamentary region and of the surrounding region are *σ*_fil_ = 1∙10^3^ S/m and *σ*_sur_ = 1∙10^−1^ S/m, respectively. This assumption is based on the LC-AFM investigations of slightly reduced SrTiO_3_, which show that the filamentary conductivity is higher by a factor of 10^4^ than the surrounding parts^27^. Additionally, the values were adjusted in such a way that the total conductance measured in the electrodegradation experiment is in correspondence with the density of dislocations measured by the etch pit technique.

In the simulation, periodic boundary conditions are chosen in the *x*-direction. Thus, the model mimics the situation of periodic filaments with a spacing of the chosen width, *w*. One of the two electrode contacts is set to a constant voltage of *V*_appl_ = 30 V, while the other is set to ground. As the metal electrodes are considered infinite heat sinks, the temperature at these boundaries is set to *T*_0_ = 700 K. Because there is no current and heat flow out of the top and bottom electrode, Neumann boundaries are used at these.

**Figure 4:**
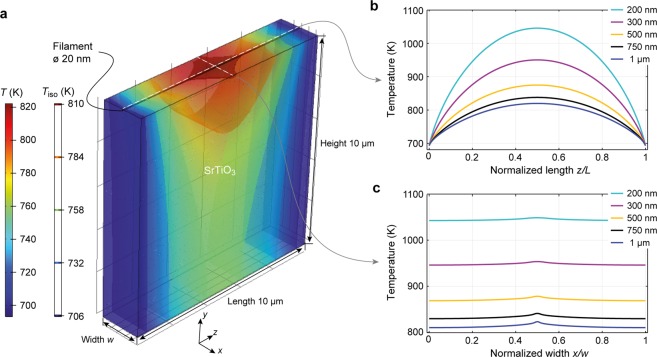
Finite element simulation: (**a**) simulation geometry for a width of *w* = 1 µm. The filament diameter is 20 nm. In *x-*direction periodic boundary conditions are assumed, meaning that filaments are periodically placed with distance *w*. The simulated temperature *T* is encoded in colour. In addition, five different isosurfaces *T*_iso_ are shown. The temperature at the surfaces along the filament axis and perpendicular to it are displayed in (**b**,**c**), respectively, for the different width *w*. The cut lines used to extract the data in (**b**,**c**) are indicated in (**a**) as dashed lines.

To simulate the influence of filament density on temperature distribution during the electroreduction process, the width of the slab is varied from *w* = 1 µm down to *w* = 200 nm. This would be equivalent to an increase in the dislocation density by a factor of 5. Figure 4a shows the temperature distribution on the surface and temperature isosurfaces within the slab for *w* = 1 µm. It can be clearly seen that the hottest spot appears in the middle of the filament. Figure 4b,c shows the temperature at the surface along the filament and perpendicular to the filament, respectively. Along the filament, a parabolic temperature distribution evolves, as the heat is only dissipated via the two electrodes. When increasing the filament density by reducing the width, the total temperature increases (Fig. 4b). In addition, the temperature distribution at the surface perpendicular to the filament becomes more homogenous (Fig. 4c), which means that even if the measured surface temperature appears to be homogenous, the source of the temperature increase can be the current flow through a network of small filaments/dislocations. It should be noted that this phenomenological model is not intended to make assumptions about the origin of the increased conductivity along of dislocations, which is still under debate. Some models predict that dislocations have a higher initial conductivity due to space charge effects^40^ while other models attribute the increased conductivity to the preferential generation of oxygen vacancies at the core of dislocations^36^ leading to a localized valence change of the Ti ion within the course of electroreduction. Either way, filamentary current paths located at dislocations would be present and thus this has been the input for our model. The obtained simulation results are in agreement with the experimental electroreduction data. In the beginning, the dislocation density is lower and the filaments/dislocations appear hotter than the surroundings due to the confined current. Nevertheless, the temperature is increased close to the dislocations, enabling further reduction processes and the possible formation of additional dislocations/filaments. When the filament/dislocation density increases, the overall temperature appears more homogenous at the surface, even though the Joule heating is localized. This is consistent with experimental observations. Note that a homogenous electronic conductivity was assumed in the simulations, leading to a parabolic temperature distribution along the filament axis. If an inhomogeneous electronic conductivity is assumed, the hot spot would shift to the more resistive region at the anode due to stoichiometry polarization, as observed in the experiment.

## Conclusion

In summary, we have shown that extended defects such as dislocations and bundles of dislocations can serve as thermally- and electrically-conducting channels during the electroreduction of SrTiO_3_. In the first stage of degradation are activated the already present dislocations, which have formed due to the preparation, i.e., by sawing or scratching, serving as preferential guides for the current flow. After prolonged electroreduction, the matrix of the sample also undergoes a transformation and the region of highest electrically-induced reduction can be found within the shortest connection between the anode and cathode, whereas a hotspot is formed on the anode side. This stage of electroreduction is tightly connected to the gliding of existing dislocations and, moreover, with the evolution of additional dislocations due to thermal and electrical stress. The 3D electro-thermal simulation results support the increase of the dislocation density during electroreduction. The model confirms that thin conduction channels can lead to effective heating of the sample. Moreover, the model predicts the homogenization of the temperature at the sample surface with the increase of the filament/dislocation density, which is consistent with the experimental observations. The presented experiments, underlining the prominent role of dislocations during electroreduction, can be regarded as a kind of emulation of resistive switching properties and hence could lead to further understanding of the mechanism, which is needed for the development of future applications. Regarding memristive devices, a controlled manipulation of the position of the dislocations could allow for a predetermination of the filament position and could therefore be a possible route to overcoming endurance and reliability issues.

## Methods

Single crystals of undoped, Verneuil-grown SrTiO_3_ (Crystec, Berlin, Germany) with epi-polished (100) surface have been investigated. At first 3 × 10 × 0.5 mm^3^ crystals with epi-polished surface were electroreduced and compared with crystals with a surface roughened by mechanical scratching using a diamond tip. The electrodes were applied afterwards using Pt paste (Demetron, Hanau, Germany). In a second experiment, only one defined scratch was introduced manually using a diamond tip on a 10 × 10 × 0.5 mm^3^ crystal in order to increase the dislocation density locally. Here, 30 nm thick Pt electrodes of 5 × 3 mm^2^ size, separated by 3 mm, were deposited by sputtering. The electrodes were contacted to a voltage source via metallic wires being gently pressed to the surface by mechanical springs. The actual geometry of this setup manufactured in-house is illustrated in Fig. 2a. To avoid contaminations and to establish an ambient acting as oxygen sink for the crystal, electroreduction experiments were conducted under UHV conditions (*p* < 10^−6^ mbar) and maintained by a turbomolecular pump (Leybold, Cologne, Germany). For each electroreduction experiment, the sample was positioned on a heating element and heated up to temperatures of 350–400 °C, which accelerated the electroreduction process by slightly activating the electronic and ionic conductivity. The voltage (200 V max.) was applied using a voltage source (6430, Keithley, Solon, USA), providing internal current compliance at 10 mA. For application of high voltages (1 kV max.), an external voltage source (BOP 1000 M, Kepco, Flushing, USA) was combined with a series resistor of 10 MΩ, also resulting in a current compliance of 10 mA. The temperature evolution was monitored by a forward-looking infrared (FLIR) camera (X6540sc, FLIR Systems, Täby, Sweden) with an emissivity of 0.45. The infrared camera was calibrated for the SrTiO_3_ surface beforehand using a thermocouple as reference. Luminescence intensity and lifetime imaging upon two-photon excitation (λ_exc_ = 740 nm) was performed on a laser scanning microscope (A1R, Nikon, Amsterdam, The Netherlands), as described previously^35,41^. FEM simulations were performed using COMSOL Multiphysics (COMSOL, Stockholm, Sweden).

## Supplementary information

Supplementary Information

## Data Availability

The datasets generated during and/or analysed during the current study are available from the corresponding author on reasonable request.
