# Supplementary material for: Current channeling along extended defects during electroreduction of SrTiO3
Source: Sci Rep. 2019 Feb 21;9:2502. doi: 10.1038/s41598-019-39372-2 (PMC6385180; doi:10.1038/s41598-019-39372-2)
Supplement: Supplementary file 1 — Supplementary Information [file 41598_2019_39372_MOESM1_ESM.docx]

**Supplementary Information to**

**Current channeling along extended defects during electroreduction of SrTiO_3_**

Christian Rodenbücher^*,1^, Stephan Menzel^2,3^, Dominik Wrana^2,3,4^, Thomas Gensch^5^, Carsten Korte^1^, Franciszek Krok^4^, and Krzysztof Szot^2,3,6^

^1^ Forschungszentrum Jülich GmbH, Institute of Energy and Climate Research (IEK-3), 52425 Jülich, Germany

^2^ Forschungszentrum Jülich GmbH, Peter Grünberg Institute (PGI-7), 52425 Jülich, Germany

^3^ Forschungszentrum Jülich GmbH, JARA-FIT, 52425 Jülich, Germany

^4^ Jagiellonian University, Marian Smoluchowski Institute of Physics, 30-348 Krakow, Poland

^5^ Forschungszentrum Jülich GmbH, Institute of Complex Systems (ICS-4), 52425 Jülich, Germany

^6^ University of Silesia, A. Chełkowski Institute of Physics, 40-007 Katowice, Poland

**Alignment of dislocations in SrTiO_3_**

The mechanical properties of SrTiO_3_ and the structure of its dislocation network have been investigated intensively. Gumbsch et al. have found that SrTiO_3_ single crystals can be deformed by compression below 1050 K and they concluded that $\left\langle110 \right\rangle\left\{ 1\bar{1}0 \right\}$ is the primary slip system for $\left( 001 \right)$ oriented crystals under uniaxial compression^1–3^. In recent years, the details of plastic deformation of SrTiO_3_ have been elucidated further using nanoindentation experiments combined with transition electron microscopy and etch pits analysis uncovering the additional slip systems $\left\langle110 \right\rangle\left\{ 110 \right\}$ and $\left\langle110 \right\rangle\left\{ 001 \right\}$ ^4,5^. Simulations employing molecular dynamics even indicated a slip system $\left\langle100 \right\rangle\left\{ 001 \right\}$ parallel to the surface and thus invisible for investigations using etch pits techniques^6^. Although the presence of this variety of different slip systems allows for the evolution of a complex network of dislocations, $\left\langle110 \right\rangle\left\{ 1\bar{1}0 \right\}$ appears to be the most prominent slip system leading to the evolution of the characteristic dislocation pile ups observed on SrTiO_3_ $\left( 001 \right)$ single crystal surfaces. Using nanoindentation measurements, it was demonstrated that pile ups oriented along $\left\langle100 \right\rangle$ direction related to dislocations lying in $\left\{ 110 \right\}_{45^{\circ}}$ planes as well as oriented along $\left\langle110 \right\rangle$ direction related to dislocations lying in $\left\{ 110 \right\}_{90^{\circ}}$ planes evolve^6^. The dislocations pile ups oriented along $\left\langle100 \right\rangle$ arrange more easily than the $\left\langle110 \right\rangle$ pile ups and can be induced even by gentle mechanical stress such as by pressing the crystal with sharp tweezers and can be thus found on epi-polished SrTiO_3_ surfaces frequently^7^.

In the main text, we have presented detailed investigations of the generation of dislocations of the unscratched sample upon electroreduction. Two effects have been found by etching experiments marking the exits of dislocations on the surface, i) an agglomeration of dislocations in $\left\langle100 \right\rangle$ direction in between the electrodes close to the anode and ii) an agglomeration of dislocations at the rim of the sample with preferential orientation in $\left\langle110 \right\rangle$ direction which was attributed to the generation of strain related to the formation of the hotspot at the anode (Fig. 3). In the following, we intend to give evidence that the observed dislocations indeed evolved during the electroreduction and are not an artefact induced e.g. induced during handling the crystals with tweezers. Therefore we conducted etch pits experiments on SrTiO_3_ single crystals obtained from the same manufacturer as those used for the electroreduction experiments in the main text. At first, we marked the exits of dislocations of the as received surface using hydrofluoric acid. Subsequently we applied slight mechanical stress by touching and pressing the crystal using tweezers to simulate typical sample handling before etching the crystal again to detect whether a significant result of dislocations has been induced in this way. As shown in Fig. S1 this is not the case. The sizes of the etch pits have been increased as a consequence of the repeated etching but the distribution of etch pits before and after tweezers handling is almost identical. The magnifications obtained at one corner of a crystal reveal that the majority of etch pits is distributed randomly but there are some agglomerations showing pile ups in $\left\langle100 \right\rangle$ direction. Those pile ups are characteristic for as received crystal surfaces and may be related to mechanical stress during polishing and transport.


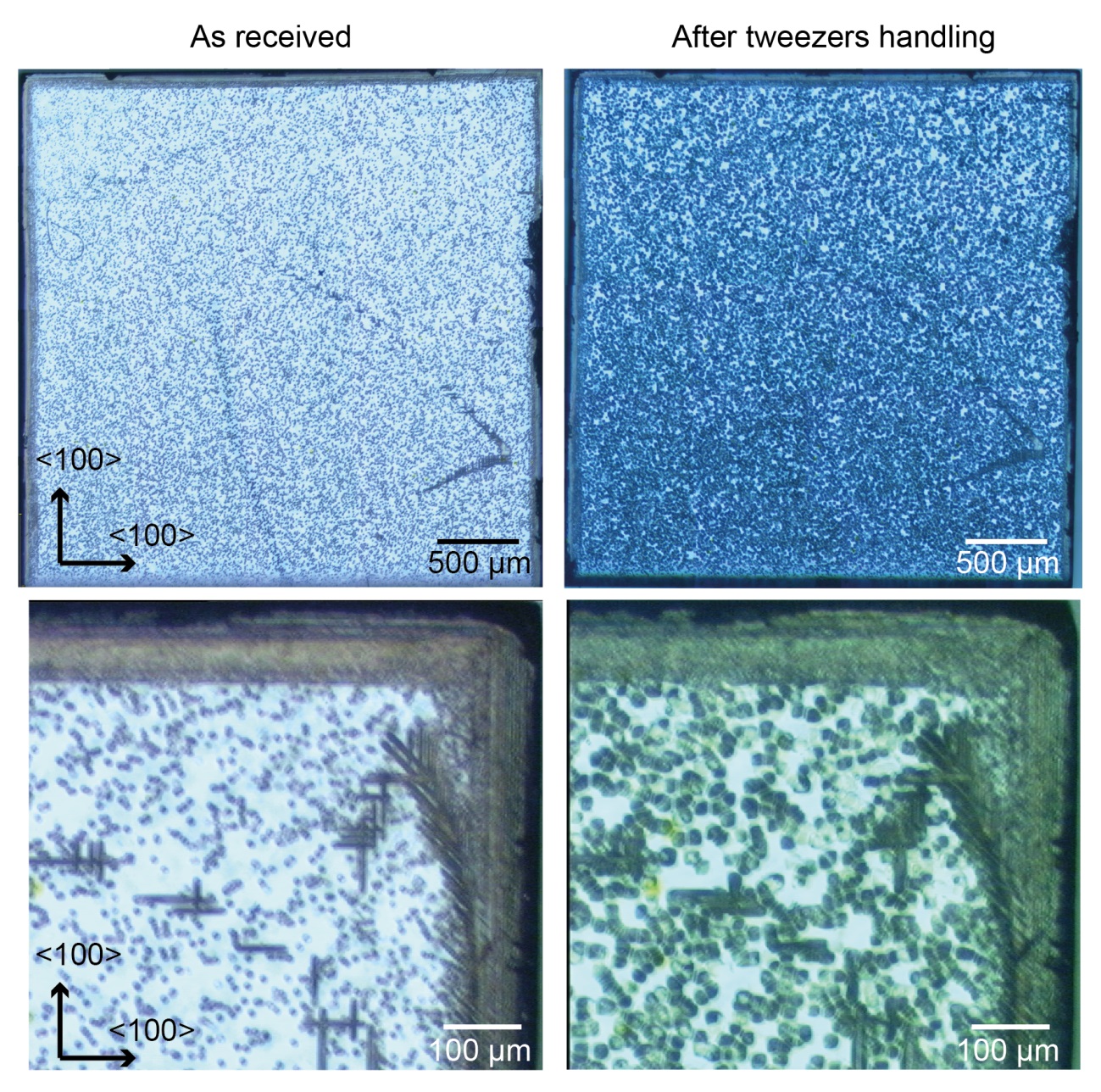


Fig. S1: Etch pits analysis of the SrTiO_3_ (100) surface by optical phase contrast microscopy. The distribution of etch pits of the as received (left) and the crystal handled extensively by tweezers (right) is nearly identical.

Also at the rim of the crystal where the density of dislocations is significantly induced due to the cutting by wire saw, most of the etch pits are oriented along $\left\langle100 \right\rangle$ but some indications for pile ups in $\left\langle110 \right\rangle$ direction can be seen. This supports the conclusion that pile ups along $\left\langle100 \right\rangle$ can be induced more easily than those in $\left\langle110 \right\rangle$ direction. Most importantly, in the as received crystals (Fig. S1), we did not observe any pre-existing dislocation pile ups with such high density as in the crystals after electroreduction (Fig. 3b). Hence, we conclude that both, the pile ups in between the electrodes as well as the pile ups connected to the hotspot at the anode have been created during electrodegradation. Taking into account that during electroreduction oxygen is transported towards the anode which can lead to local pressures about 60 MPa as seen when investigating the bubble formation at the SrTiO_3_/Pt interface at the anode^8^, it is plausible to assume that the evolution of the hot spot at the anode led to the evolution of large internal strain which was sufficient to form even dislocations on $\left\{ 110 \right\}_{90^{\circ}}$ planes being identified as $\left\langle110 \right\rangle$ etch pits pile ups. This is further supported regarding the phase contrast microscopy analysis (Fig. 3a) showing that the dislocation-rich region at the rim is directly connected to the hot spot via a strained zone visible as dark contrast showing that there is a close connection between the electroreduction behaviour and the mechanical properties of SrTiO_3_.

**References**

1. Gumbsch, P., Taeri-Baghbadrani, S., Brunner, D., Sigle, W. & Rühle, M. Plasticity and an Inverse Brittle-to-Ductile Transition in Strontium Titanate. *Phys. Rev. Lett.* **87,** 085505 (2001).

2. Brunner, D., Taeri-Baghbadrani, S., Sigle, W. & Rühle, M. Surprising Results of a Study on the Plasticity in Strontium Titanate. *J. Am. Ceram. Soc.* **84,** 1161–1163 (2001).

3. Yang, K., Ho, N. & Lu, H. Plastic Deformation of 〈001〉Single Crystal SrTiO_3_ by Compression at Room Temperature. *J. Am. Ceram. Soc.* **94,** 3104–3111 (2011).

4. Matsunaga, T. & Saka, H. Transmission electron microscopy of dislocations in SrTiO _3_. *Philos. Mag. Lett.* **80,** 597–604 (2000).

5. Yang, K.-H., Ho, N.-J. & Lu, H.-Y. Deformation Microstructure in (001) Single Crystal Strontium Titanate by Vickers Indentation. *J. Am. Ceram. Soc.* **92,** 2345–2353 (2009).

6. Javaid, F., Stukowski, A. & Durst, K. 3D Dislocation structure evolution in strontium titanate: Spherical indentation experiments and MD simulations. *J. Am. Ceram. Soc.* **100,** 1134–1145 (2017).

7. Kamaladasa, R. J. *et al.* Dislocation impact on resistive switching in single-crystal SrTiO_3_. *J. Appl. Phys.* **113,** 234510 (2013).

8. Wojtyniak, M. *et al.* Electro-degradation and resistive switching of Fe-doped SrTiO 3 single crystal. *J. Appl. Phys.* **113,** 083713 (2013).
